# Supplementary material for: Disorder of Sex Development Due to 17-Beta-Hydroxysteroid Dehydrogenase Type 3 Deficiency: A Case Report and Review of 70 Different HSD17B3 Mutations Reported in 239 Patients
Source: Int J Mol Sci. 2022 Sep 2;23(17):10026. doi: 10.3390/ijms231710026 (PMC9456484; doi:10.3390/ijms231710026)
Supplement: Supplementary file 1 [file ijms-23-10026-s001.zip › ijms-1813632-supplementary.pdf]

Supplementary Table S1. Published germline mutations of the *HSD17B3* gene.

Mutation [a]	Predicted Effect on Protein	Mutation Type [b]	Zygosity[c]	Number of affected family members	Origin	Reference
**Exon 1**						
c.74_75delTG	p.(Val25Glufs*54)	fs	Compound Het. with exon 1 deletion	1	China	[1]
c.128_131delTCTT	p.(Phe43Cysfs*9)	fs	Hom.	1	China	[2]
c.133C>T	p.(Arg45Trp)	ms	Compound Het. with c.645A>T	1	n/a	[3]
c.133C>T	p.(Arg45Trp)	ms	Hom.	1	UK	[4]
c.133C>T	p.(Arg45Trp)	ms	Compound Het. with c.277+4A>T	1	UK	[4]
c.133C>T	p.(Arg45Trp)	ms	Compound Het. with c.694_698delins	1	France	[5]
c.139A>G	p.(Met47Val)	ms	Compound Het. with c.645A>T	1	n/a	[3]
c.139A>G	p.(Met47Val)	ms	Hom.	1	n/a	[3]
c.139A>G	p.(Met47Val)	ms	Compound Het. with p.(V243fs)	1	Ukraine	[6]
c.150G>A	p.(Trp50*)	ns	Hom.	1	Egypt	[7]
c.152C>T	p.(Ala51Val)	ms	Compound Het. with c.527A>C	1	Egypt	[8]
**Exon 2**						
c.160A>G	p.(Thr54Ala)	ms	Hom.	1	Egypt	[8]
c.160A>G	p.(Thr54Ala)	ms	Hom.	1	Egypt	[9]
c.160A>G	p.(Thr54Ala)	ms	Hom.	1	Turkey	[10]
c.160A>G	p.(Thr54Ala)	ms	Hom.	1	Turkey	[11]
c.166G>A	p.(Ala56Thr)	ms	Hom.	1	UK	[12]
c.166G>A	p.(Ala56Thr)	ms	Hom.	2	UK	[13]
c.166G>A	p.(Ala56Thr)	ms	Hom.	1	UK	[14]
c.179T>C	p.(Ile60Thr)	ms	Hom.	1	China	[1]
c.179T>C	p.(Ile60Thr)	ms	Compound Het. with exon 1 deletion	1	China	[1]
c.191A>G	p.(Tyr64Cys)	ms	Hom.	1	n/a	[3]
c.194C>T	p.(Ser65Leu)	ms	Hom.	1	Iran	[15]
c.194C>T	p.(Ser65Leu)	ms	Compound Het. with c.239G>A	1	US	[16]
c.194C>T	p.(Ser65Leu)	ms	Compound Het. with c.729_735del	1	UK	[4]
c.194C>T	p.(Ser65Leu)	ms	Hom.	1	Iran	[17]
c.201+1G>A	p.(?)	sp	Compound Het. with c.277+5G>A	3	UK	[14]
**Exon 3**						
c.202-1G>A	p.(?)	sp	Compound Het. with c.277+4A>T	1	UK	[14]
c.203T>G	p.(Leu68Arg)	ms	Compound Het. with c.277+4A>T	1	UK	[14]
c.210delA	p.(Lys70Asnfs*16)	fs	Hom.	1	n/a	[3]
c.221A>C	p.(Asn74Thr)	ms	Hom.	1	The Netherlands	[18]
c.221A>C	p.(Asn74Thr)	ms	Hom.	1	The Netherlands	[18]
c.221A>C	p.(Asn74Thr)	ms	Compound Het. with c.277+4A>T	2	The Netherlands	[18]

Mutation [a]	Predicted Effect on Protein	Mutation Type [b]	Zygosity[c]	Number of affected family members	Origin	Reference
c.221A>C	p.(Asn74Thr)	ms	Compound Het. with c.277+4A>T	1	The Netherlands	[18]
c.238C>T	p.(Arg80Trp)	ms	Hom.	1	Spain	[19]
c.238C>T	p.(Arg80Trp)	ms	Hom.	1	Italy	[20]
c.238C>T	p.(Arg80Trp)	ms	Compound Het. with c.608C>A	1	Italy	[20]
c.238C>T	p.(Arg80Trp)	ms	Hom.	1	Lebanon	[21]
c.238C>T	p.(Arg80Trp)	ms	Hom.	3	Iran	[22]
c.238C>T	p.(Arg80Trp)	ms	Compound Het. with c.277+4A>T	1	US	[23]
c.238C>T	p.(Arg80Trp)	ms	Hom.	1	n/a	[3]
c.238C>T	p.(Arg80Trp)	ms	Hom.	3	Saudi Arabia	[24]
c.239G>T	p.(Arg80Leu)	ms	Compound Het. with c.839T>C	2	China	[25]
c.239G>T	p.(Arg80Leu)	ms	Compound Het. with c.389A>G	1	Germany	[26]
c.239G>A	p.(Arg80Gln)	ms	Hom.	1	Palestine	[27]
c.239G>A	p.(Arg80Gln)	ms	Compound Het. with 278-1G>C	1	Brazil	[27]
c.239G>A	p.(Arg80Gln)	ms	Hom.	1	Brazil	[15]
c.239G>A	p.(Arg80Gln)	ms	Hom.	8	Israel	[28]
c.239G>A	p.(Arg80Gln)	ms	Hom.	2	Israel	[28]
c.239G>A	p.(Arg80Gln)	ms	Hom.	1	Israel	[28]
c.239G>A	p.(Arg80Gln)	ms	Hom.	1	Israel	[28]
c.239G>A	p.(Arg80Gln)	ms	Hom.	1	Israel	[28]
c.239G>A	p.(Arg80Gln)	ms	Hom.	1	Israel	[28]
c.239G>A	p.(Arg80Gln)	ms	Hom.	5	Israel	[28]
c.239G>A	p.(Arg80Gln)	ms	Hom.	1	Israel	[28]
c.239G>A	p.(Arg80Gln)	ms	Hom.	1	Israel	[28]
c.239G>A	p.(Arg80Gln)	ms	Hom.	1	The Netherlands	[18]
c.239G>A	p.(Arg80Gln)	ms	Compound Het. with c.277+4A>T	1	Germany	[29]
c.239G>A	p.(Arg80Gln)	ms	Hom.	1	Brazil	[30]
c.239G>A	p.(Arg80Gln)	ms	Compound Het. with duplication exons 3-10	1	Cyprus	[31]
c.239G>A	p.(Arg80Gln)	ms	Compound Het. with c.130-2A>G (*)	1	Italy	[32]
c.239G>A	p.(Arg80Gln)	ms	Compound Het. with c.614T>A	1	US	[23]
c.239G>A	p.(Arg80Gln)	ms	Compound Het. with c.194C>T	1	US	[16]
c.239G>A	p.(Arg80Gln)	ms	Hom.	1	Turkey	[33]
c.239G>A	p.(Arg80Gln)	ms	Hom.	2	Brazil	[34]
c.239G>A	p.(Arg80Gln)	ms	Compound Het. with 278-1G>C	1	Brazil	[34]
c.239G>A	p.(Arg80Gln)	ms	Hom.	1	Turkey	[10]
c.239G>A	p.(Arg80Gln)	ms	Compound Het. with c.277G>A	1	Turkey	[10]
c.239G>A	p.(Arg80Gln)	ms	Hom.	1	Turkey	[11]
c.239G>A	p.(Arg80Gln)	ms	Compound Het. with c.277G>A	2	Turkey	[11]
c.239G>A	p.(Arg80Gln)	ms	Hom.	1	Turkey	[35]
c.239G>A	p.(Arg80Gln)	ms	Compound Het. with c.257_265del	1	Italy	[36]
c.257_265delAGGCCATTG	p.(Glu86_Ile88del)	if	Compound Het. with c.239G>A	1	Italy	[36]
c.277G>A	p.(Glu93Lys)	ms	Compound Het. with c.239G>A	1	Turkey	[10]

Mutation [a]	Predicted Effect on Protein	Mutation Type [b]	Zygosity[c]	Number of affected family members	Origin	Reference
c.277G>A	p.(Glu93Lys)	ms	Compound Het. with c.239G>A	2	Turkey	[11]
c.277+2T>G	p.(?)	sp	Compound Het. with c.277+4A>T	1	Brazil	[30]
c.277+2T>G	p.(?)	sp	Compound Het. with c.277+4A>T	1	Brazil	[37]
c.277+4A>T	p.(?)	sp	Hom.	1	US	[15]
c.277+4A>T	p.(?)	sp	Hom.	1	US	[15]
c.277+4A>T	p.(?)	sp	Hom.	1	Germany	[15]
c.277+4A>T	p.(?)	sp	Compound Het. with c.845C>T	1	US	[15]
c.277+4A>T	p.(?)	sp	Compound Het. with c.527A>C	1	US	[15]
c.277+4A>T	p.(?)	sp	Hom.	2	The Netherlands	[18]
c.277+4A>T	p.(?)	sp	Hom.	1	The Netherlands	[18]
c.277+4A>T	p.(?)	sp	Hom.	1	The Netherlands	[18]
c.277+4A>T	p.(?)	sp	Hom.	1	The Netherlands	[18]
c.277+4A>T	p.(?)	sp	Compound Het. with c.221A>C	2	The Netherlands	[18]
c.277+4A>T	p.(?)	sp	Compound Het. with c.221A>C	1	The Netherlands	[18]
c.277+4A>T	p.(?)	sp	Compound Het. with c.239G>A	1	The Netherlands	[18]
c.277+4A>T	p.(?)	sp	Compound Het. with c.239G>A	1	The Netherlands	[18]
c.277+4A>T	p.(?)	sp	Compound Het. with 278-1G>C	1	The Netherlands	[18]
c.277+4A>T	p.(?)	sp	Compound Het. with 278-1G>C	1	The Netherlands	[18]
c.277+4A>T	p.(?)	sp	Compound Het. with c.845C>T	1	The Netherlands	[18]
c.277+4A>T	p.(?)	sp	Hom.	1	Germany	[29]
c.277+4A>T	p.(?)	sp	Hom.	1	Germany	[29]
c.277+4A>T	p.(?)	sp	Hom.	1	Germany	[29]
c.277+4A>T	p.(?)	sp	Compound Het. with c.239G>A	1	Germany	[29]
c.277+4A>T	p.(?)	sp	Hom.	1	Germany	[38]
c.277+4A>T	p.(?)	sp	Hom.	1	US	[39]
c.277+4A>T	p.(?)	sp	Compound Het. with c.608C>T	1	Italy	[20]
c.277+4A>T	p.(?)	sp	Compound Het. with c.277+2T>G	1	Brazil	[30]
c.277+4A>T	p.(?)	sp	Compound Het. with c.238C>T	1	US	[23]
c.277+4A>T	p.(?)	sp	Compound Het. with duplication exons 3-10	2	US	[40]
c.277+4A>T	p.(?)	sp	Compound Het. with c.203T>G	1	UK	[14]

Mutation [a]	Predicted Effect on Protein	Mutation Type [b]	Zygosity[c]	Number of affected family members	Origin	Reference
c.277+4A>T	p.(?)	sp	Compound Het. with c.608C>T	1	UK	[14]
c.277+4A>T	p.(?)	sp	Compound Het. with c.845C>T	1	UK	[14]
c.277+4A>T	p.(?)	sp	Compound Het. with c.202-1G>A	1	UK	[14]
c.277+4A>T	p.(?)	sp	Compound Het. with c.673G>A	1	UK	[14]
c.277+4A>T	p.(?)	sp	Compound Het. with c.278-1G>C	2	UK	[14]
c.277+4A>T	p.(?)	sp	Compound Het. with c.278-1G>C	2	UK	[14]
c.277+4A>T	p.(?)	sp	Hom.	1	UK	[14]
c.277+4A>T	p.(?)	sp	Hom.	1	UK	[14]
c.277+4A>T	p.(?)	sp	Compound Het. with c.277+2T>G	1	Brazil	[37]
c.277+4A>T	p.(?)	sp	Hom.	1	Germany	[26]
c.277+4A>T	p.(?)	sp	Hom.	1	Germany	[26]
c.277+4A>T	p.(?)	sp	Compound Het. with c.389A>G	1	Germany	[26]
c.277+4A>T	p.(?)	sp	Compound Het. with c.645A>T	1	UK	[4]
c.277+4A>T	p.(?)	sp	Compound Het. with c.133C>T	1	UK	[4]
c.277+4A>T	p.(?)	sp	Compound Het. with c.640_645del	1	Italy	[41]
c.277+4A>T	p.(?)	sp	Compound Het. with c.902C>T	1	France	[5]
c.277+4A>T	p.(?)	sp	Compound Het. with c.645A>T	1	France	[42]
c.277+4A>T	p.(?)	sp	Compound Het. with p.(Glu215Asp)	1	Ukraine	[6]
c.277+4A>T	p.(?)	sp	Hom.	2	Denmark	[43]
c.277+5G>A	p.(?)	sp	Compound Het. with c.201+1G>A	3	UK	[14]
**Exon 4**						
278-1G>C	p.(?)	sp	Compound Het. with c.239G>A	1	Brazil	[27]
278-1G>C	p.(?)	sp	Compound Het. with c.277+4A>T	1	The Netherlands	[18]
278-1G>C	p.(?)	sp	Compound Het. with c.277+4A>T	1	The Netherlands	[18]
278-1G>C	p.(?)	sp	Hom.	1	The Netherlands	[18]
278-1G>C	p.(?)	sp	Hom.	1	Brazil	[44]
278-1G>C	p.(?)	sp	Hom.	1	Brazil	[44]
278-1G>C	p.(?)	sp	Compound Het. with c.277+4A>T	2	UK	[14]
278-1G>C	p.(?)	sp	Compound Het. with c.277+4A>T	2	UK	[14]
278-1G>C	p.(?)	sp	Compound Het. with c.608C>T	2	Brazil	[34]
278-1G>C	p.(?)	sp	Hom.	1	Brazil	[34]
278-1G>C	p.(?)	sp	Hom.	1	Brazil	[34]

Mutation [a]	Predicted Effect on Protein	Mutation Type [b]	Zygosity[c]	Number of affected family members	Origin	Reference
278-1G>C	p.(?)	sp	Compound Het. with c.239G>A	1	Brazil	[34]
c.383T>C	p.(Leu128Ser)	ms	Hom.	1	n/a	[3]
**Exon 5**						
c.389A>G	p.(Asn130Ser)	ms	Compound Het. with ?	1	UK	[12]
c.389A>G	p.(Asn130Ser)	ms	Compound Het. with ?	1	The Netherlands	[18]
c.389A>G	p.(Asn130Ser)	ms	Compound Het. with c.560delT	1	Germany	[29]
c.389A>G	p.(Asn130Ser)	ms	Compound Het. with ?	1	UK	[13]
c.389A>G	p.(Asn130Ser)	ms	Compound Het. with c.599C>T	1	UK	[14]
c.389A>G	p.(Asn130Ser)	ms	Hom.	1	Canada	[45]
c.389A>G	p.(Asn130Ser)	ms	Compound Het. with c.277+4A>T	1	Germany	[26]
c.389A>G	p.(Asn130Ser)	ms	Compound Het. with c.239G>A	1	Germany	[26]
c.397G>A	p.(Gly133Arg)	ms	Compound Het. with c.618C>A	3	Tunisia	[46]
c.397G>A	p.(Gly133Arg)	ms	Compound Het. with c.618C>A	3	Tunisia	[47]
n/a	p.(L139Pfs)	fs	Hom.	1	n/a	[3]
**Exon 6**						
c.454-1G>A	p.(?)	sp	Hom.	1	US	[48]
c.464A>C	p.(His155Pro)	ms	Hom.	1	Turkey	[33]
c.464A>C	p.(His155Pro)	ms	Hom.	1	Turkey	[11]
**Exon 7**						
c.491T>C	p.(Met164Thr)	ms	Hom.	1	Egypt	[8]
c.517delG	p.(Glu173Asnfs*9)	fs	Hom.	1	Egypt	[8]
c.524G>C	p.(Arg175Thr)	ms	Hom.	1	Turkey	[10]
c.524+2T>A	p.(?)	sp	Hom.	1	Sudan	[49]
**Exon 8**						
c.527A>C	p.(Gln176Pro)	ms	Compound Het. with c.277+4A>T	1	US	[15]
c.527A>C	p.(Gln176Pro)	ms	Hom.	2	Egypt	[8]
c.527A>C	p.(Gln176Pro)	ms	Compound Het. with c.152C>T	1	Egypt	[8]
c.527A>C	p.(Gln176Pro)	ms	Compound Het. with c.618C>A	1	Tunisia	[47]
c.540C>T	p.(Ile180Ile)	syn	Compound Het. with c.581T>C	1	Egypt	[8]
c.560delT	p.(Ile187Lysfs*40)	fs	Compound Het. with c.389A>G	1	Germany	[29]
c.563C>T	p.(Ala188Val)	ms	Hom.	1	The Netherlands	[18]
c.576G>A	p.(Trp192*)	ns	Hom.	1	Oman	[50]
c.581T>C	p.(Leu194Pro)	ms	Compound Het. with c.540C>T	1	Egypt	[8]
c.590T>A	p.(Met197Lys)	ms	Hom.	1	UK	[13]
c.590T>A	p.(Met197Lys)	ms	Hom.	1	UK	[14]
c.599C>T	p.(Ala200Val)	ms	Compound Het. with c.599C>T	1	UK	[14]
**Exon 9**						
c.607-1G>A	p.(?)	sp	Hom.	1	Syria	[27]
c.607-1G>A	p.(?)	sp	Hom.	1	Greece	[15]
c.607-1G>A	p.(?)	sp	Hom.	2	Turkey	[51]

Mutation [a]	Predicted Effect on Protein	Mutation Type [b]	Zygosity[c]	Number of affected family members	Origin	Reference
c.607-1G>A	p.(?)	sp	Hom.	3	The Netherlands	[18]
c.607-1G>A	p.(?)	sp	Compound Het. with c.695C>T	1	Greece	[52]
c.608C>A	p.(Ala203Glu)	ms	Compound Het. with c.238C>T	1	Italy	[20]
c.608C>T	p.(Ala203Val)	ms	Hom.	1	Brazil	[27]
c.608C>T	p.(Ala203Val)	ms	Hom.	1	Brazil	[44]
c.608C>T	p.(Ala203Val)	ms	Compound Het. with c.277+4A>T	1	Italy	[20]
c.608C>T	p.(Ala203Val)	ms	Hom.	1	Brazil	[30]
c.608C>T	p.(Ala203Val)	ms	Compound Het. with c.625T>C	1	Brazil	[53]
c.608C>T	p.(Ala203Val)	ms	Hom.	1	Yemen	[53]
c.608C>T	p.(Ala203Val)	ms	Compound Het. with c.277+4A>T	1	UK	[14]
c.608C>T	p.(Ala203Val)	ms	Compound Het. with 278-1G>C	2	Brazil	[34]
c.608C>T	p.(Ala203Val)	ms	Hom.	1	Brazil	[34]
c.608C>T	p.(Ala203Val)	ms	Hom.	2	Brazil	[34]
c.608C>T	p.(Ala203Val)	ms	Compound Het. with c.645 A>T	1	Portugal	This study
c.614T>A	p.(Val205Glu)	ms	Compound Het. with ?	1	US	[15]
c.614T>A	p.(Val205Glu)	ms	Hom.	1	UK	[13]
c.614T>A	p.(Val205Glu)	ms	Compound Het. with c.239G>A	1	US	[23]
c.614T>A	p.(Val205Glu)	ms	Hom	1	UK	[14]
c.614T>A	p.(Val205Glu)	ms	Compound Het. with c.645A>T	1	UK	[4]
c.618C>A	p.(Cys206*)	ns	Hom.	1	Tunisia	[54]
c.618C>A	p.(Cys206*)	ns	Compound Het. with c.397G>A	3	Tunisia	[46]
c.618C>A	p.(Cys206*)	ns	Hom.	3	Tunisia	[47]
c.618C>A	p.(Cys206*)	ns	Compound Het. with c.397G>A	3	Tunisia	[47]
c.618C>A	p.(Cys206*)	ns	Compound Het. with c.527A>C	1	Tunisia	[47]
c.622T>A	p.(Phe208Ile)	ms	Hom.	1	Germany	[15]
c.625T>C	p.(Ser209Pro)	ms	Compound Het. with c.608C>T	1	Brazil	[53]
c.625T>C	p.(Ser209Pro)	ms	Hom.	1	Brazil	[34]
c.635T>A	p.(Leu212Gln)	ms	Compound Het. with c.703A>G	1	Italy	[55]
c.635T>A	p.(Leu212Gln)	ms	Compound Het. with c.703A>G	1	Italy	[20]
n/a	p.(Glu214Argfs*4)	fs	Compound Het. with c.761_762delAG	1	Turkey	[56]
c.640_645delGAGGAA	p.(Glu214_Glu215del)	if	Compound Het. with c.277+4A>T	1	Italy	[41]
n/a	p.(Glu215Asp)	ms	Hom.	1	Brazil	[15]
n/a	p.(Glu215Asp)	ms	Hom.	1	UK	[13]
n/a	p.(Glu215Asp)	ms	Hom.	1	Portugal	[57]
n/a	p.(Glu215Asp)	ms	Hom.	2	Brazil	[34]
n/a	p.(Glu215Asp)	ms	Compound Het. with c.277+4A>T	1	Ukraine	[6]
c.645A>T	p.(Glu215Asp)	ms	Compound Het. with c.139A>G	1	n/a	[3]
c.645A>T	p.(Glu215Asp)	ms	Compound Het. with c.133C>T	1	n/a	[3]

Mutation [a]	Predicted Effect on Protein	Mutation Type [b]	Zygosity[c]	Number of affected family members	Origin	Reference
c.645A>T	p.(Glu215Asp)	ms	Compound Het. with c.277+4A>T	1	UK	[4]
c.645A>T	p.(Glu215Asp)	ms	Compound Het. with c.614T>A	1	UK	[4]
c.645A>T	p.(Glu215Asp)	ms	Hom.	1	Italy	[58]
c.645A>T	p.(Glu215Asp)	ms	Compound Het. with c.277+4A>T	1	France	[42]
c.645A>T	p.(Glu215Asp)	ms	Compound Het. with c.608 C>T	1	Portugal	This study
**Exon 10**						
c.673-1G>C	p.(?)	sp	Hom.	1	Turkey	[59]
c.673G>A	p.(Val225Met)	sp	Compound Het. with c.277+4A>T	1	UK	[14]
c.673G>A	p.(Val225Met)	sp	Hom.	3	Israel	[60]
c.673G>A	p.(Val225Met)	sp	Hom.	1	Israel	[61]
c.694_698delinsCCCATA	p.(Ser232Profs*18)	fs	Compound Het. with c.133C>T	1	France	[5]
c.695C>T	p.(Ser232Leu)	ms	Compound Het. with c.703A>G	1	US	[27]
c.695C>T	p.(Ser232Leu)	ms	Hom.	3	UK	[13]
c.695C>T	p.(Ser232Leu)	ms	Compound Het. with c.607-1G>A	1	Greece	[52]
c.695C>T	p.(Ser232Leu)	ms	Hom.	1	UK	[4]
c.703A>G	p.(Met235Val)	ms	Compound Het. with c.695C>T	1	US	[27]
c.703A>G	p.(Met235Val)	ms	Compound Het. with c.635T>A	1	Italy	[55]
c.703A>G	p.(Met235Val)	ms	Compound Het. with c.635T>A	1	Italy	[20]
n/a	p.(Val243fs)	fs	Compound Het. with c.139A>G	1	Ukraine	[6]
c.729_735delGATAACC	p.(Ile244Argfs*11)	fs	Hom.	1	Poland	[15]
c.729_735delGATAACC	p.(Ile244Argfs*11)	fs	Hom.	1	Turkey	[62]
c.729_735delGATAACC	p.(Ile244Argfs*11)	fs	Hom.	1	Egypt	[8]
c.729_735delGATAACC	p.(Ile244Argfs*11)	fs	Compound Het. with c.194C>T	1	UK	[4]
c.761_762delAG	p.(Glu254Valfs*10)	fs	Compound Het. with p.(Glu214Arg.fs*4)	1	Turkey	[56]
c.761_762delAG	p.(Glu254Valfs*10)	fs	Hom.	1	Turkey	[63]
c.761_762delAG	p.(Glu254Valfs*10)	fs	Hom.	1	Syria	[35]
c.803G>A	p.(Cys268Tyr)	ms	Hom.	1	UK	[64]
c.803G>A	p.(Cys268Tyr)	ms	Hom.	5	UK	[13]
c.812A>G	p.(His271Arg)	ms	n/a	1	France	[65]
**Exon 11**						
c.839T>C	p.(Leu280Pro)	ms	Compound Het. with c.239G>T	2	China	[25]
c.845C>T	p.(Pro282Leu)	sp	Compound Het. with c.277+4A>T	1	US	[15]
c.845C>T	p.(Pro282Leu)	sp	Compound Het. with c.277+4A>T	1	The Netherlands	[18]
c.845C>T	p.(Pro282Leu)	sp	Hom.	1	Spain	[66]
c.845C>T	p.(Pro282Leu)	sp	Compound Het. with c.277+4A>T	1	UK	[14]
c.852G>A	p.(Trp284*)	ns	Hom.	2	China	[2]
c.861C>A	p.(Tyr287*)	ns	Hom.	1	Turkey	[67]
c.861C>A	p.(Tyr287*)	ns	Hom.	2	Turkey	[10]

Mutation [a]	Predicted Effect on Protein	Mutation Type [b]	Zygosity[c]	Number of affected family members	Origin	Reference
c.861C>A	p.(Tyr287*)	ns	Hom.	2	Turkey	[11]
c.902C>T	p.(Ala301Val)	ms	Compound Het. with c.277+4A>T	1	France	[5]
**Gross rearrangements**						
Gross duplication exons 3-10 (11.96 kb)	p.(?)		Compound Het. with c.239G>A	1	Cyprus	[31]
Gross duplication exons 3-10 (25.4 kb)	p.(?)		Compound Het. with c.277+4A>T	2	US	[40]
Gross deletion exon 1 (461 bp)	p.(?)		Hom.	1	US	[16]
Gross deletion exon 1	p.(?)		Compound Het. with c.74_75delTG	1	China	[1]
Gross deletion exon 1	p.(?)		Compound Het. with c.179T>C	1	China	[1]

[a] Published *HSD17B3* germline mutations were identified by searching the PubMed database (National Center for Biotechnology Information, U.S. National Library of Medicine, National Institutes of Health) (https://www.ncbi.nlm.nih.gov/pubmed) for articles, using the keywords "mutation" combined with "*HSD17B3*" or "17 beta hydroxysteroid dehydrogenase 3". Reference lists of articles were also searched to identify further articles. Publications were analyzed for relevant content and a total of 68 articles reported one or more patients with *HSD17B3* mutations. Articles were analyzed for evidence of duplication of data and patients that had been included in previous mutation studies were excluded from the analysis. Each published mutation was checked for accuracy by comparison to the *HSD17B3* wild-type sequence. Errors due to the incorrect assignment of nucleotide or codon numbers or translation errors between codon and amino acid residues were corrected whenever possible. Mutations shown only at the amino acid level were converted to single-nucleotide changes when it was possible to predict the altered base using the genetic code. When more than one nucleotide change could account for the amino acid change or when other ambiguous changes were indicated, the precise mutation was considered unavailable. The numbering of each nucleotide was changed, whenever necessary, to comply with current recommendations for mutation nomenclature [68], whereby nucleotide +1 was the A of the ATG-translation initiation codon. Mutations were described in relation to the *HSD17B3* cDNA reference sequence (GenBank accession number NM_000197.2).
[b] Mutation type: fs, frameshift; ns, nonsense; ms, missense; if, in-frame deletion or insertion; sp, splice site; syn, synonymous.
[c] Zygosity: Hom, homozygous; Het, heterozygous.
n/a, not available.
(*) discordant numbering.

References

1. Yu, B.; Liu, Z.; Mao, J.; Wang, X.; Zheng, J.; Xiong, S.; Cui, M.; Ma, W.; Huang, Q.; Xu, H., et al. Novel mutations of HSD17B3 in three Chinese patients with 46,XY Disorders of Sex Development. *Steroids* **2017**, *126*, 1-6, doi:10.1016/j.steroids.2017.07.009.

2. Yang, Z.; Ye, L.; Wang, W.; Zhao, Y.; Wang, W.; Jia, H.; Dong, Z.; Chen, Y.; Wang, W.; Ning, G., et al. 17beta-Hydroxysteroid dehydrogenase 3 deficiency: Three case reports and a systematic review. *J Steroid Biochem Mol Biol* **2017**, *174*, 141-145, doi:10.1016/j.jsbmb.2017.08.012.

3. Eggers, S.; Sadedin, S.; van den Bergen, J.A.; Robevska, G.; Ohnesorg, T.; Hewitt, J.; Lambeth, L.; Bouty, A.; Knarston, I.M.; Tan, T.Y., et al. Disorders of sex development: insights from targeted gene sequencing of a large international patient cohort. *Genome Biol* **2016**, *17*, 243, doi:10.1186/s13059-016-1105-y.

4. Hughes, L.A.; McKay-Bounford, K.; Webb, E.A.; Dasani, P.; Clokie, S.; Chandran, H.; McCarthy, L.; Mohamed, Z.; Kirk, J.M.W.; Krone, N.P., et al. Next generation sequencing (NGS) to improve the diagnosis and management of patients with disorders of sex development (DSD). *Endocr Connect* **2019**, *8*, 100-110, doi:10.1530/EC-18-0376.

5. Ea, V.; Bergougnoux, A.; Philibert, P.; Servant-Fauconnet, N.; Faure, A.; Breaud, J.; Gaspari, L.; Sultan, C.; Paris, F.; Kalfa, N. How Far Should We Explore Hypospadias? Next-generation Sequencing Applied to a Large Cohort of Hypospadiac Patients. *Eur Urol* **2021**, *79*, 507-515, doi:10.1016/j.eururo.2020.12.036.

6. Globa, E.; Zelinska, N.; Shcherbak, Y.; Bignon-Topalovic, J.; Bashamboo, A.; Msmall es, C.K. Disorders of Sex Development in a Large Ukrainian Cohort: Clinical Diversity and Genetic Findings. *Front Endocrinol (Lausanne)* **2022**, *13*, 810782, doi:10.3389/fendo.2022.810782.

7. Hassan, H.A.; Mazen, I.; Gad, Y.Z.; Ali, O.S.; Mekkawy, M.; Essawi, M.L. A novel nonsense mutation in exon 1 of HSD17B3 gene in an Egyptian 46,XY adult female presenting with primary amenorrhea. *Sex Dev* **2013**, *7*, 277-281, doi:10.1159/000351822.

8. Hassan, H.A.; Mazen, I.; Gad, Y.Z.; Ali, O.S.; Mekkawy, M.; Essawi, M.L. Mutational Profile of 10 Afflicted Egyptian Families with 17-beta-HSD-3 Deficiency. *Sex Dev* **2016**, *10*, 66-73, doi:10.1159/000445311.

9. Engeli, R.T.; Tsachaki, M.; Hassan, H.A.; Sager, C.P.; Essawi, M.L.; Gad, Y.Z.; Kamel, A.K.; Mazen, I.; Odermatt, A. Biochemical Analysis of Four Missense Mutations in the HSD17B3 Gene Associated With 46,XY Disorders of Sex Development in Egyptian Patients. *J Sex Med* **2017**, *14*, 1165-1174, doi:10.1016/j.jsxm.2017.07.006.

10. Ozen, S.; Onay, H.; Atik, T.; Solmaz, A.E.; Ozkinay, F.; Goksen, D.; Darcan, S. Rapid Molecular Genetic Diagnosis with Next-Generation Sequencing in 46,XY Disorders of Sex Development Cases: Efficiency and Cost Assessment. *Horm Res Paediatr* **2017**, *87*, 81-87, doi:10.1159/000452995.

11. Ata, A.; Ozen, S.; Onay, H.; Uzun, S.; Goksen, D.; Ozkinay, F.; Ozbaran, N.B.; Ulman, I.; Darcan, S. A large cohort of disorders of sex development and their genetic characteristics: 6 novel mutations in known genes. *Eur J Med Genet* **2021**, *64*, 104154, doi:10.1016/j.ejmg.2021.104154.

12. Moghrabi, N.; Hughes, I.A.; Dunaif, A.; Andersson, S. Deleterious missense mutations and silent polymorphism in the human 17beta-hydroxysteroid dehydrogenase 3 gene (HSD17B3). *J Clin Endocrinol Metab* **1998**, *83*, 2855-2860, doi:10.1210/jcem.83.8.5052.

13. Lee, Y.S.; Kirk, J.M.; Stanhope, R.G.; Johnston, D.I.; Harland, S.; Auchus, R.J.; Andersson, S.; Hughes, I.A. Phenotypic variability in 17beta-hydroxysteroid dehydrogenase-3 deficiency and diagnostic pitfalls. *Clin Endocrinol (Oxf)* **2007**, *67*, 20-28, doi:10.1111/j.1365-2265.2007.02829.x.

14. Phelan, N.; Williams, E.L.; Cardamone, S.; Lee, M.; Creighton, S.M.; Rumsby, G.; Conway, G.S. Screening for mutations in 17beta-hydroxysteroid dehydrogenase and androgen receptor in women presenting with partially virilised 46,XY disorders of sex development. *Eur J Endocrinol* **2015**, *172*, 745-751, doi:10.1530/EJE-14-0994.

15. Andersson, S.; Geissler, W.M.; Wu, L.; Davis, D.L.; Grumbach, M.M.; New, M.I.; Schwarz, H.P.; Blethen, S.L.; Mendonca, B.B.; Bloise, W., et al. Molecular genetics and pathophysiology of 17 beta-hydroxysteroid dehydrogenase 3 deficiency. *J Clin Endocrinol Metab* **1996**, *81*, 130-136, doi:10.1210/jcem.81.1.8550739.

16. Baxter, R.M.; Arboleda, V.A.; Lee, H.; Barseghyan, H.; Adam, M.P.; Fechner, P.Y.; Bargman, R.; Keegan, C.; Travers, S.; Schelley, S., et al. Exome sequencing for the diagnosis of 46,XY disorders of sex development. *J Clin Endocrinol Metab* **2015**, *100*, E333-344, doi:10.1210/jc.2014-2605.

17. Rafigh, M.; Salmaninejad, A.; Sorouri Khorashad, B.; Arabi, A.; Milanizadeh, S.; Hiradfar, M.; Abbaszadegan, M.R. Novel Deleterious Mutation in Steroid-5alpha-Reductase-2 in 46, XY Disorders of Sex Development: Case Report Study. *Fetal Pediatr Pathol* **2022**, *41*, 141-148, doi:10.1080/15513815.2020.1745974.

18. Boehmer, A.L.; Brinkmann, A.O.; Sandkuijl, L.A.; Halley, D.J.; Niermeijer, M.F.; Andersson, S.; de Jong, F.H.; Kayserili, H.; de Vroede, M.A.; Otten, B.J., et al. 17Beta-hydroxysteroid dehydrogenase-3 deficiency: diagnosis, phenotypic variability, population genetics, and worldwide distribution of ancient and de novo mutations. *J Clin Endocrinol Metab* **1999**, *84*, 4713-4721, doi:10.1210/jcem.84.12.6174.

19. Bilbao, J.R.; Loridan, L.; Audi, L.; Gonzalo, E.; Castano, L. A novel missense (R80W) mutation in 17-beta-hydroxysteroid dehydrogenase type 3 gene associated with male pseudohermaphroditism. *Eur J Endocrinol* **1998**, *139*, 330-333, doi:10.1530/eje.0.1390330.

20. Bertelloni, S.; Balsamo, A.; Giordani, L.; Fischetto, R.; Russo, G.; Delvecchio, M.; Gennari, M.; Nicoletti, A.; Maggio, M.C.; Concolino, D., et al. 17beta-Hydroxysteroid dehydrogenase-3 deficiency: from pregnancy to adolescence. *J Endocrinol Invest* **2009**, *32*, 666-670, doi:10.1007/BF03345738.

21. George, M.M.; Sinha, S.; Mamkin, I.; Philibert, P.; New, M.I.; Wilson, R.C.; Sultan, C.; Ten, S.; Bhangoo, A. Isolated mild clitoral hypertrophy may reveal 46,XY disorders of sex development in infancy due to 17betaHSD-3 defect confirmed by molecular analysis. *Gynecol Endocrinol* **2011**, *27*, 890-894, doi:10.3109/09513590.2010.544134.

22. Omrani, M.D.; Adamovic, T.; Grandell, U.; Saleh-Gargari, S.; Nordenskjold, A. 17-beta-hydroxysteroid dehydrogenase type 3 deficiency in three adult Iranian siblings. *Sex Dev* **2011**, *5*, 273-276, doi:10.1159/000335006.

23. Chuang, J.; Vallerie, A.; Breech, L.; Saal, H.M.; Alam, S.; Crawford, P.; Rutter, M.M. Complexities of gender assignment in 17beta-hydroxysteroid dehydrogenase type 3 deficiency: is there a role for early orchiectomy? *Int J Pediatr Endocrinol* **2013**, *2013*, 15, doi:10.1186/1687-9856-2013-15.

24. Alswailem, M.; Alsagheir, A.; Abbas, B.B.; Alzahrani, O.; Alzahrani, A.S. Molecular genetics of disorders of sex development in a highly consanguineous population. *J Steroid Biochem Mol Biol* **2021**, *208*, 105736, doi:10.1016/j.jsbmb.2020.105736.

25. Wu, S.; Zheng, B.; Liu, T.; Zhu, Z.; Gu, W.; Liu, Q. [17 beta-hydroxysteroid dehydrogenase 3 deficiency due to novel compound heterozygous variants of HSD17B3 gene in a sib pair]. *Zhonghua Yi Xue Yi Chuan Xue Za Zhi* **2021**, *38*, 787-790, doi:10.3760/cma.j.cn511374-20200527-00392.

26. Hiort, O.; Marshall, L.; Birnbaum, W.; Wunsch, L.; Holterhus, P.M.; Dohnert, U.; Werner, R. Pubertal Development in17Beta-Hydroxysteroid Dehydrogenase Type 3 Deficiency. *Horm Res Paediatr* **2017**, *87*, 354-358, doi:10.1159/000453613.

27. Geissler, W.M.; Davis, D.L.; Wu, L.; Bradshaw, K.D.; Patel, S.; Mendonca, B.B.; Elliston, K.O.; Wilson, J.D.; Russell, D.W.; Andersson, S. Male pseudohermaphroditism caused by mutations of testicular 17 beta-hydroxysteroid dehydrogenase 3. *Nat Genet* **1994**, *7*, 34-39, doi:10.1038/ng0594-34.

28. Rosler, A.; Silverstein, S.; Abeliovich, D. A (R80Q) mutation in 17 beta-hydroxysteroid dehydrogenase type 3 gene among Arabs of Israel is associated with pseudohermaphroditism in males and normal asymptomatic females. *J Clin Endocrinol Metab* **1996**, *81*, 1827-1831, doi:10.1210/jcem.81.5.8626842.

29. Twesten, W.; Holterhus, P.; Sippell, W.G.; Morlot, M.; Schumacher, H.; Schenk, B.; Hiort, O. Clinical, endocrine, and molecular genetic findings in patients with 17beta-hydroxysteroid dehydrogenase deficiency. *Horm Res* **2000**, *53*, 26-31, doi:10.1159/000023509.

30. Castro, C.C.; Guaragna-Filho, G.; Calais, F.L.; Coeli, F.B.; Leal, I.R.; Cavalcante-Junior, E.F.; Monlleo, I.L.; Pereira, S.R.; Silva, R.B.; Gabiatti, J.R., et al. Clinical and molecular spectrum of patients with 17beta-hydroxysteroid dehydrogenase type 3 (17-beta-HSD3) deficiency. *Arq Bras Endocrinol Metabol* **2012**, *56*, 533-539, doi:10.1590/s0004-27302012000800012.

31. Neocleous, V.; Sismani, C.; Shammas, C.; Efstathiou, E.; Alexandrou, A.; Ioannides, M.; Argyrou, M.; Patsalis, P.C.; Phylactou, L.A.; Skordis, N. Duplication of exons 3-10 of the HSD17B3 gene: a novel type of genetic defect underlying 17beta-HSD-3 deficiency. *Gene* **2012**, *499*, 250-255, doi:10.1016/j.gene.2012.03.031.

32. Russo, G.; di Lascio, A.; Ferrario, M.; Meroni, S.; Hiort, O.; Chiumello, G. 46,XY karyotype in a female phenotype fetus: a challenging diagnosis. *J Pediatr Adolesc Gynecol* **2012**, *25*, e77-79, doi:10.1016/j.jpag.2012.03.001.

33. Demir, K.; Yildiz, M.; Elmas, O.N.; Korkmaz, H.A.; Tunc, S.; Olukman, O.; Hazan, F.; Ozkan, K.U.; Ozkan, B. Two different patterns of mini-puberty in two 46,XY newborns with 17beta-hydroxysteroid dehydrogenase type 3 deficiency. *J Pediatr Endocrinol Metab* **2015**, *28*, 961-965, doi:10.1515/jpem-2014-0365.

34. Mendonca, B.B.; Gomes, N.L.; Costa, E.M.; Inacio, M.; Martin, R.M.; Nishi, M.Y.; Carvalho, F.M.; Tibor, F.D.; Domenice, S. 46,XY disorder of sex development (DSD) due to 17beta-hydroxysteroid dehydrogenase type 3 deficiency. *J Steroid Biochem Mol Biol* **2017**, *165*, 79-85, doi:10.1016/j.jsbmb.2016.05.002.

35. Manyas, H.; Eroglu Filibeli, B.; Ayranci, I.; Guvenc, M.S.; Dundar, B.N.; Catli, G. Early and late diagnoses of 17beta-Hydroxysteroid dehydrogenase type-3 deficiency in two unrelated patients. *Andrologia* **2021**, *53*, e14017, doi:10.1111/and.14017.

36. Cocchetti, C.; Baldinotti, F.; Romani, A.; Ristori, J.; Mazzoli, F.; Vignozzi, L.; Maggi, M.; Fisher, A.D. A Novel Compound Heterozygous Mutation of HSD17B3 Gene Identified in a Patient With 46,XY Difference of Sexual Development. *Sex Med* **2022**, *10*, 100522, doi:10.1016/j.esxm.2022.100522.

37. de Calais, F.L.; Smith, L.D.; Raponi, M.; Maciel-Guerra, A.T.; Guerra-Junior, G.; de Mello, M.P.; Baralle, D. A study of splicing mutations in disorders of sex development. *Sci Rep* **2017**, *7*, 16202, doi:10.1038/s41598-017-16296-3.

38. Twesten, W.; Johannisson, R.; Holterhus, P.M.; Hiort, O. [Severe 46,XY virilization deficit due to 17beta-hydroxysteroid dehydrogenase deficiency]. *Klin Padiatr* **2002**, *214*, 314-315, doi:10.1055/s-2002-34015.

39. Mains, L.M.; Vakili, B.; Lacassie, Y.; Andersson, S.; Lindqvist, A.; Rock, J.A. 17beta-hydroxysteroid dehydrogenase 3 deficiency in a male pseudohermaphrodite. *Fertil Steril* **2008**, *89*, 228 e213-227, doi:10.1016/j.fertnstert.2007.02.048.

40. Massanyi, E.Z.; Gearhart, J.P.; Kolp, L.A.; Migeon, C.J. Novel mutation among two sisters with 17beta hydroxysteroid dehydrogenase type 3 deficiency. *Urology* **2013**, *81*, 1069-1071, doi:10.1016/j.urology.2012.12.024.

41. Faienza, M.F.; Baldinotti, F.; Marrocco, G.; TyuTyusheva, N.; Peroni, D.; Baroncelli, G.I.; Bertelloni, S. 17beta-hydroxysteroid dehydrogenase type 3 deficiency: female sex assignment and follow-up. *J Endocrinol Invest* **2020**, *43*, 1711-1716, doi:10.1007/s40618-020-01248-y.

42. Garcia, A.; Legendre, M.; Chantot-Bastaraud, S.; Siffroi, J.P.; Christin-Maitre, S. Unravelling a case of 46,XY DSD due to 17ss-Hydroxysteroid Dehydrogenase type 3 mutations at the age of 49. *Ann Endocrinol (Paris)* **2022**, 10.1016/j.ando.2022.01.003, doi:10.1016/j.ando.2022.01.003.

43. von Spreckelsen, B.; Aksglaede, L.; Johannsen, T.H.; Nielsen, J.E.; Main, K.M.; Jorgensen, A.; Jensen, R.B. Prepubertal and pubertal gonadal morphology, expression of cell lineage markers and hormonal evaluation in two 46,XY siblings with 17beta-hydroxysteroid dehydrogenase 3 deficiency. *J Pediatr Endocrinol Metab* **2022**, 10.1515/jpem-2021-0713, doi:10.1515/jpem-2021-0713.

44. Mendonca, B.B.; Inacio, M.; Arnhold, I.J.; Costa, E.M.; Bloise, W.; Martin, R.M.; Denes, F.T.; Silva, F.A.; Andersson, S.; Lindqvist, A., et al. Male pseudohermaphroditism due to 17 beta-hydroxysteroid dehydrogenase 3 deficiency. Diagnosis, psychological evaluation, and management. *Medicine (Baltimore)* **2000**, *79*, 299-309, doi:10.1097/00005792-200009000-00003.

45. Grimbly, C.; Caluseriu, O.; Metcalfe, P.; Jetha, M.M.; Rosolowsky, E.T. 46,XY disorder of sex development due to 17-beta hydroxysteroid dehydrogenase type 3 deficiency: a plea for timely genetic testing. *Int J Pediatr Endocrinol* **2016**, *2016*, 12, doi:10.1186/s13633-016-0030-x.

46. Engeli, R.T.; Rhouma, B.B.; Sager, C.P.; Tsachaki, M.; Birk, J.; Fakhfakh, F.; Keskes, L.; Belguith, N.; Odermatt, A. Biochemical analyses and molecular modeling explain the functional loss of 17beta-hydroxysteroid dehydrogenase 3 mutant G133R in three Tunisian patients with 46, XY Disorders of Sex Development. *J Steroid Biochem Mol Biol* **2016**, *155*, 147-154, doi:10.1016/j.jsbmb.2015.10.023.

47. Ben Rhouma, B.; Kallabi, F.; Mahfoudh, N.; Ben Mahmoud, A.; Engeli, R.T.; Kamoun, H.; Keskes, L.; Odermatt, A.; Belguith, N. Novel cases of Tunisian patients with mutations in the gene encoding 17beta-hydroxysteroid dehydrogenase type 3 and a founder effect. *J Steroid Biochem Mol Biol* **2017**, *165*, 86-94, doi:10.1016/j.jsbmb.2016.03.007.

48. Mueller, O.T.; Coovadia, A. Novel human pathological mutations. Gene symbol: HSD17B3. Disease: 17 beta-hydroxysteroid dehydrogenase-3 deficiency. *Hum Genet* **2009**, *125*, 335.

49. Ellaithi, M.; Werner, R.; Riepe, F.G.; Krone, N.; Kulle, A.E.; Diab, T.; Kamel, A.K.; Arlt, W.; Holterhus, P.M.; Sabir, O., et al. 46,XY disorder of sex development in a sudanese patient caused by a novel mutation in the HSD17B3 gene. *Sex Dev* **2014**, *8*, 151-155, doi:10.1159/000363201.

50. Al-Sinani, A.; Mula-Abed, W.A.; Al-Kindi, M.; Al-Kusaibi, G.; Al-Azkawi, H.; Nahavandi, N. A Novel Mutation Causing 17-beta-Hydroxysteroid Dehydrogenase Type 3 Deficiency in an Omani Child: First Case Report and Review of Literature. *Oman Med J* **2015**, *30*, 129-134, doi:10.5001/omj.2015.27.

51. Can, S.; Zhu, Y.S.; Cai, L.Q.; Ling, Q.; Katz, M.D.; Akgun, S.; Shackleton, C.H.; Imperato-McGinley, J. The identification of 5 alpha-reductase-2 and 17 beta-hydroxysteroid dehydrogenase-3 gene defects in male pseudohermaphrodites from a Turkish kindred. *J Clin Endocrinol Metab* **1998**, *83*, 560-569, doi:10.1210/jcem.83.2.4535.

52. Galli-Tsinopoulou, A.; Serbis, A.; Kotanidou, E.P.; Litou, E.; Dokousli, V.; Mouzaki, K.; Fanis, P.; Neocleous, V.; Skordis, N. 46,XY Disorder of Sex Development due to 17-Beta Hydroxysteroid Dehydrogenase Type 3 Deficiency in an Infant of Greek Origin. *J Clin Res Pediatr Endocrinol* **2018**, *10*, 74-78, doi:10.4274/jcrpe.4829.

53. Khattab, A.; Yuen, T.; Yau, M.; Domenice, S.; Frade Costa, E.M.; Diya, K.; Muhuri, D.; Pina, C.E.; Nishi, M.Y.; Yang, A.C., et al. Pitfalls in hormonal diagnosis of 17-beta hydroxysteroid dehydrogenase III deficiency. *J Pediatr Endocrinol Metab* **2015**, *28*, 623-628, doi:10.1515/jpem-2014-0295.

54. Ben Rhouma, B.; Belguith, N.; Mnif, M.F.; Kamoun, T.; Charfi, N.; Kamoun, M.; Abdelhedi, F.; Hachicha, M.; Kamoun, H.; Abid, M., et al. A novel nonsense mutation in HSD17B3 gene in a Tunisian patient with sexual ambiguity. *J Sex Med* **2013**, *10*, 2586-2589, doi:10.1111/j.1743-6109.2012.02763.x.

55. Bertelloni, S.; Maggio, M.C.; Federico, G.; Baroncelli, G.; Hiort, O. 17beta-hydroxysteroid dehydrogenase-3 deficiency: a rare endocrine cause of male-to-female sex reversal. *Gynecol Endocrinol* **2006**, *22*, 488-494, doi:10.1080/09513590600921358.

56. Sagsak, E.; Aycan, Z.; Savas-Erdeve, S.; Keskin, M.; Cetinkaya, S.; Karaer, K. 17betaHSD-3 enzyme deficiency due to novel mutations in the HSD17B3 gene diagnosed in a neonate. *J Pediatr Endocrinol Metab* **2015**, *28*, 957-959, doi:10.1515/jpem-2014-0354.

57. Costa, C.; Castro-Correia, C.; Mira-Coelho, A.; Monteiro, B.; Monteiro, J.; Hughes, I.; Fontoura, M. The dilemma of the gender assignment in a Portuguese adolescent with disorder of sex development due to 17beta-hydroxysteroid-dehydrogenase type 3 enzyme deficiency. *Endocrinol Diabetes Metab Case Rep* **2014**, *2014*, 140064, doi:10.1530/EDM-14-0064.

58. De Falco, L.; Piscopo, C.; D'Angelo, R.; Evangelista, E.; Suero, T.; Sirica, R.; Ruggiero, R.; Savarese, G.; Di Carlo, A.; Furino, G., et al. Detection of 46, XY Disorder of Sex Development (DSD) Based on Plasma Cell-Free DNA and Targeted Next-Generation Sequencing. *Genes (Basel)* **2021**, *12*, doi:10.3390/genes12121890.

59. Ciftci, N.; Kayas, L.; Camtosun, E.; Akinci, A. 46,XY Sex Development Defect due to a Novel Homozygous (Splice Site) c.673_1G>C Variation in the HSD17B3 Gene: Case Report. *J Clin Res Pediatr Endocrinol* **2022**, *14*, 233-238, doi:10.4274/jcrpe.galenos.2020.2020.0249.

60. Levy-Khademi, F.; Zeligson, S.; Lavi, E.; Klopstock, T.; Chertin, B.; Avnon-Ziv, C.; Abulibdeh, A.; Renbaum, P.; Rosen, T.; Perlberg-Bengio, S., et al. The novel founder homozygous V225M mutation in the HSD17B3 gene causes aberrant splicing and XY-DSD. *Endocrine* **2020**, *69*, 650-654, doi:10.1007/s12020-020-02327-z.

61. Bertalan, R.; Admoni, O.; Bashamboo, A.; Tenenbaum-Rakover, Y.; McElreavey, K. A novel HSD17B3 gene mutation in a 46,XY female-phenotype newborn identified by whole-exome sequencing. *Clin Endocrinol (Oxf)* **2017**, *87*, 407-408, doi:10.1111/cen.13396.

62. Alikasifoglu, A.; Hiort, O.; Gonc, N.; Demirbilek, H.; Isik, E.; Kandemir, N. 17beta-hydroxysteroid dehydrogenase type 3 deficiency as a result of a homozygous 7 base pair deletion in 17betaHSD3 gene. *J Pediatr Endocrinol Metab* **2012**, *25*, 561-563, doi:10.1515/jpem-2012-0009.

63. Alikasifoglu, A.; Vuralli, D.; Hiort, O.; Gonc, N.; Ozon, A.; Kandemir, N. Severe Undervirilisation in a 46,XY Case Due to a Novel Mutation in HSD17B3 Gene. *J Clin Res Pediatr Endocrinol* **2015**, *7*, 249-252, doi:10.4274/jcrpe.2069.

64. Lindqvist, A.; Hughes, I.A.; Andersson, S. Substitution mutation C268Y causes 17 beta-hydroxysteroid dehydrogenase 3 deficiency. *J Clin Endocrinol Metab* **2001**, *86*, 921-923, doi:10.1210/jcem.86.2.7172.

65. George, M.M.; New, M.I.; Ten, S.; Sultan, C.; Bhangoo, A. The clinical and molecular heterogeneity of 17betaHSD-3 enzyme deficiency. *Horm Res Paediatr* **2010**, *74*, 229-240, doi:10.1159/000318004.

66. Bel, J.; Mainou, A.; Blanco, J.A.; Bilbao, J.R. [Male pseudohermaphroditism due to 17 beta-hydroxysteroid dehydrogenase deficiency]. *Med Clin (Barc)* **2003**, *120*, 795, doi:10.1016/s0025-7753(03)73848-2.

67. Tuhan, H.U.; Anik, A.; Catli, G.; Ceylaner, S.; Dundar, B.; Bober, E.; Abaci, A. A novel missense mutation in HSD17B3 gene in a 46, XY adolescent presenting with primary amenorrhea and virilization at puberty. *Clin Chim Acta* **2015**, *438*, 154-156, doi:10.1016/j.cca.2014.07.025.

68. den Dunnen, J.T.; Dalgleish, R.; Maglott, D.R.; Hart, R.K.; Greenblatt, M.S.; McGowan-Jordan, J.; Roux, A.F.; Smith, T.; Antonarakis, S.E.; Taschner, P.E. HGVS Recommendations for the Description of Sequence Variants: 2016 Update. *Hum Mutat* **2016**, *37*, 564-569, doi:10.1002/humu.22981.
